# Significant contribution of chronotype to emotional well-being in chronic psychiatric outpatients in Greece

**DOI:** 10.1016/j.nsa.2024.103940

**Published:** 2024-01-11

**Authors:** Eva-Maria Tsapakis, Konstantinos N. Fountoulakis, Stefania Kanioura, Haim Einat

**Affiliations:** a Faculty of Medicine, Aristoteles University, Thessaloniki, Greece; b School of Behavioral Sciences, The Academic College of Tel-Aviv-Yaffo, Tel-Aviv, Israel

**Keywords:** Chronobiology, Psychiatry, Emotional well-being, Sleep

## Abstract

Circadian rhythms are pivotal for human functioning, and their disruption holds significant implications for well-being. One common source for circadian disruptions is circadian misalignment that can be related to chronotypes. Chronotypes refer to an individual's preferred timing for sleep and wakefulness. Individuals with late chronotypes are at a disadvantage in the morning-oriented modern world and are demonstrated to have negative consequences in many aspects of life. In the context of psychiatric disorders, chronotypes are related to prevalence of disorders and to treatment effects but less attention is given to the relationship between chronotype and well-being in chronic psychiatric patients. The current study aims to elucidate the extent to which individual chronotypes contribute to emotional well-being within a cohort of individuals with chronic psychiatric disorder in outpatient clinic.

Participants (n = 100) were recruited from the outpatient clinic of the 3rd Department of Psychiatry, AHEPA University Hospital, Thessaloniki, Greece, and the AX Mental Health Outpatient Clinic in Heraklion, Crete, Greece. Most participants were diagnosed within the F2 cluster, Psychotic disorders (n = 38), or F3 cluster, Mood (affective) disorders (n = 48). The Morningness-Eveningness Questionnaire (MEQ) was employed to assess chronotype. The STAI-Y1, CES-D, QoL Uniscale and RASS Questionnaires were used to assess aspects of emotional well-being. A single measure of emotional well-being was generated using Z-score transformation. Student's t-test, ANOVAs and Pearson's correlations were used to identify parameters contributing to emotional well-being, followed by a comprehensive regression model.

Results show a significant contribution to emotional well-being by “psychiatric diagnosis” with schizophrenia/schizoaffective patients showing better emotional well-being compared with the “other” group, “receiving treatment” with patients receiving treatment showing higher score than ones who do not receive treatment, and “morningness/eveningness preference” where morningness was associated with higher score of emotional well-being. No other demographic or health-related parameters were significantly associated with emotional well-being score.

These findings clearly indicate the critical importance of chronotypes to the emotional well-being of chronic psychiatric patients. Additional thought and research should explore possible chronotherapy interventions that will address this issue in patients.

## Highlights

• Chronotypes (morningness/eveningness preference) are factors in psychiatric disorders.
• We explored the contribution of chronotypes to emotional well-being in psychiatric patients.
• Results show a significant contribution of chronotypes, similar in magnitude to diagnosis.
• We suggest that chronotherapy intervention may be of assistance to patients.

## 1 Introduction

Circadian rhythms are central to human life and disruptions to these rhythms are associated with significant negative consequences in biology and behavior, in health, and disease (Turek, 1994; Karatsoreos et al., 2011; Zimmet et al., 2019). Circadian disruptions were strongly associated with all major psychiatric disorders as well as with sub-clinical psychiatry-related symptoms. Multiple studies show that disrupted circadian rhythms and ensuing sleep disturbances increase the risk for psychiatric disorders (Delorme et al., 2020; Tapia-Osorio et al., 2013; Ng et al., 2015), are detected in psychiatric patients more than in the general population (Zou et al., 2022), and are implicated as a core symptom for at least some major psychiatric disorders (Delorme et al., 2020; McClung, 2007; Ritter et al., 2012).

One common source for circadian disruptions is circadian misalignment, a term that describes a variety of circumstances where the individual's biological rhythms are not synchronized with their schedules (Baron et al., 2014). Circadian misalignment can be found in different contexts such as shift work (Boivin et al., 2022), jetlag (Bin et al., 2019), delayed sleep phase syndrome (Zisapel, 2001) and social jetlag (Roenneberg, 2023). Of these, social jetlag and delayed sleep phase syndrome are strongly related to the concept of chronotypes.

Chronotypes refer to an individual's preferred timing for sleep and wakefulness, reflecting their inherent biological predisposition to be more alert and active at certain times of the day. Whereas the morningness/eveningness preference is a continuous trait, chronotypes categorize people into distinct groups based on their sleep-wake patterns and preferences. There are three primary chronotypes: morning types (larks), evening types (owls), and intermediate types (Roenneberg et al., 2003). Because the modern world is biased towards morning activity in both schools and most workplaces, individuals with late, evening chronotype, find themselves at a disadvantage and are forced to live much of their life in a state of circadian misalignment between their innate tendency (waking up late) and the demands of the society (attend school or work in the morning). This chronic challenge was demonstrated to have significant negative consequences in many aspects of life including school performance (Zerbini et al., 2017), physical performance (Vitale et al., 2017), productivity at work (Shimura et al., 2022), cognition (Schmidt et al., 2007), response to stress (Rofe et al., 2023), and more. Whereas many studies demonstrate the problems associated with evening chronotypes there might also be some positive outcomes. For example, an interesting study demonstrated higher emotional intelligence in evening types, expressed in greater ability to perceive and understand the emotions of others (Stolarski et al., 2015).

In the context of psychiatry, significant evidence demonstrate higher prevalence of evening chronotypes in mood disorders, including major depression, bipolar disorder and seasonal affective disorder, in drug dependence and in eating disorders (Kivelä et al., 2018; Melo et al., 2017). Data regarding anxiety disorders and psychotic disorders are less clear with some positive and some negative findings (Zou et al., 2022; Kivelä et al., 2018; Linke et al., 2021; Ahn et al., 2008). Moreover, disrupted sleep that might be related to chronotypes (Roenneberg et al., 2003) is a core symptom of depression, bipolar disorder, and anxiety disorders (Sadock et al., 2007). Furthermore, chronotypes are also related to efficacy of treatment. For example, morningness was found to be higher in lithium responders compared with non-responders bipolar patients (McCarthy et al., 2019) and response rate of bipolar patients to mood stabilizers was demonstrated to be lower in evening chronotypes compared with morning or intermediate individuals (Menculini et al., 2023). Similarly, morningness was associated with better response to antidepressants in depressed patients (Xavier et al., 2023). Yet, in partial contrast to these findings, other studies did not find a relationship between chronotype and treatment response (Wang et al., 2023) and in a study of combined antidepressants and chronotherapeutic interventions (sleep deprivation + light therapy) in depressed patients, the response was better and the response rate higher in individuals with evening chronotype (Dallaspezia et al., 2018). However, the last study included chronotherapeutic intervention which is an additional highly relevant factor.

Whereas some aspects of the relationship between chronotypes and psychiatric disorders had been studied during the last decades, less attention is given to the effects of chronotype on the life and well-being of chronic psychiatric patients living in the community. Well-being in patients with major psychiatric disorders is highly related to both the severity of symptoms and the response to treatment, but the studies mentioned above, mostly examined hospitalized patients or patients during the first weeks of medication. Hence, the current study is designed to further explore the possible relationship between chronotypes and well-being in individuals suffering from chronic psychiatric disorders and treated in an outpatient clinic. Because of the strong relationship demonstrated in the past between chronotypes and affective disorders, we specifically explored emotional aspects of well-being.

## 2 Materials and methods

### 2.1 Participants

The study included 100 participants recruited from the outpatient clinic of the 3rd Department of Psychiatry, AHEPA University Hospital in Thessaloniki, Greece, and the AX Mental Health Outpatient Clinic in Heraklion. Crete, Greece. Participants were approached by a researcher in the clinics, after routine visits, and were asked to participate in the study. Participants that agreed received oral explanation from the researcher as to the nature and general goals of the study and signed an informed consent form. The entire study was approved by the Ethics Committee of Aristotle University of Macedonia.

The inclusion criteria were the following, 1) A psychiatric diagnosis from the ICD-10 [excluding those attributed to head injury, brain damage, or substance abuse], 2) Age between 18 and 65 years old, 3) Fluent Greek speakers. From these participants, 49 were male and 51 were female, with ages ranging from 20 to 64 years (M = 42.0, SD = 12.6). Most patients were diagnosed independently within the F2 cluster, Psychotic disorders (n = 38), or F3 cluster, Mood (affective) disorders (n = 48). Additional 14 patients had various other diagnoses including obsessive compulsive disorder and unspecified disorder of adult personality and behavior.

### 2.2 Procedure

A researcher (A.K., a psychologist) approached potential participants after a routine visit to an outpatient clinic. Following informed consent, participants entered the study comprising of a Google Forms questionnaire administered by the researcher in a private room at the outpatient clinic.

### 2.3 Tools and measures

The protocol included the registration of sociodemographic and general health data. The assessment of emotional well-being was performed through 4 different aspects: (1) anxious cognition using the state part of the State-Trait Anxiety Inventory Y-1 form (STAI-Y1) a commonly used measure of trait and state anxiety (Fountoulakis et al., 2006; Spielberger, 1970, 1979); (2) negative emotionality using the Center for Epidemiologic Studies Depression Scale (CES-D) rating the frequency of experiencing symptoms associated with depression (Fountoulakis et al., 2001; Radloff, 1977); (3) self-reported life satisfaction, using the of Quality of Life Uniscale (QoL) reporting how one is satisfied from life compared to what one desires (Sloan et al., 1998; Liu et al., 2015); and (4) suicidality, using the Risk Assessment Suicidality Scale (RASS) assessing suicidal risk in the general population as well as in mental patient (Fountoulakis et al., 2012; Nadareishvili et al., 2022). Circadian preference was evaluated using the Morningness-Eveningness Questionnaire (MEQ) assessing the degree to which respondents are active and alert at certain times of day (Horne et al., 1976).

### 2.4 Data analysis

To analyze the data, we initially generated a single variable representing a combined construct of emotional well-being. This new variable combined the 4 measures of emotional well-being mentioned above using Z-Scores. The variables Self-reported life satisfaction (QoL Uniscale), Negative emotionality (CES-D questionnaire), Anxious cognition (STAI-Y questionnaire), and Suicidality (RASS questionnaire) were transformed to Z-scores using the standard formula: Z-score (x) = (x-mean)/STD. After the initial transformation, scores were all converted so that higher score indicates better well-being. Finally, the Z-scores of all variables were summed to create a new variable representing a broader perspective of emotional well-being. Pearson's correlation, Students' t-test and Analysis of variance (followed by Sceffe post-hoc tests when needed) were used to explore possible links between emotional well-being and morningness/eveningness preference (or chronotypes) and between emotional well-being and demographic and health variables. Variables that were indicated by the initial analysis as possibly linked to emotional well-being were further analyzed in an integrative stepwise, backward regression model. Normal distribution was assessed with the Shapiro-Wilk test. All statistical analyses were conducted using Statistica 13.0 (TIBCO Software Inc.).

## 3 Results

### 3.1 Demographic variables and emotional well-being

None of the demographic variables were linked with emotional well-being (Table 1): Gender – *t*-test, t (98) = 0.20, p = 0.84. Age – Pearson's correlation, r = 0.09, p = 0.39. Personal status – ANOVA, F (3,96) = 0.36, p = 0.78. Education – *t*-test, t (98) = 0.84, p = 0.40. Employment – ANOVA – F (2,97) = 0.87, p = 0.42.

**Table 1:** Demographics.

Demographic variable	Distribution
Age	Mean ± STD = 42.0 ± 12.6
	Minimum = 20.3, Maximum = 64.3
Gender	Females – 51, Males 49.
Personal status	Married or partnership – 18
	Unmarried, divorced, widowed - 82
Education	High-school or less – 53
	Tertiary education - 47
Employment	Employed – 31
	Unemployed – 59
	Students - 10

### 3.2 Health, health behavior and emotional well-being

Data for health and health behavior parameters are presented in Table 2. There is a significant link between the variable “General Health in the Last month” and emotional well-being [ANOVA – F (4,95) = 14.54, p < 0.001; post-hoc - Great/Very Good different than Bad/Mediocre; Good different than Bad; effect size (eta squared) = 0.38]. No links are shown between BMI and emotional well-being [Pearson's correlation – r = 0.03, p = 0.77] or between smoking and well-being [*t*-test, t (98) = -0.77, p = 0.44]. A possible relationship is demonstrated between alcohol consumption and well-being [ANOVA, F (3,96) = 2.82, p = 0.043], but post-hoc analysis shows the difference stems from the heavy drinking individuals that are different than other groups. However, as the number of heavy drinking participants in this sample is only 3, we suggest that with the small number of participants in the group this is not a meaningful result.

**Table 2:** Health and health behavior.

Health variable	Distribution
General health last month	Great or Very good – 33
	Good – 24
	Mediocre or bad - 43
BMI	Mean ± STD = 28.4 ± 5.5; range 17.1–44.6
	Underweight – 1; Healthy/normal – 26; Overweight – 39; Obese - 34
Smoking	Yes – 58, No-42
Alcohol consumption	Never or rarely – 69; Moderately/socially – 28; Heavy - 3

Regarding mental health, the most frequent diagnoses in this cohort were psychotic disorders and affective/anxiety disorders. Most participants were treated with antipsychotics and many treated with antidepressants (Table 3). Psychiatric diagnosis and treatment were linked with emotional well-being for original diagnosis, current diagnosis, receiving any treatment, and use of antipsychotic medications, but not use of antidepressants (Table 4).

**Table 3:** Mental health background.

Mental health variable	Distribution
Original diagnosis	Psychotic disorders – 31
	Depression/anxiety – 46
	Other - 23
Current diagnosis	Schizophrenia or Schizoaffective – 38
	Mood & anxiety disorders – 48
	Other - 14
Antipsychotics	Yes – 76, No - 24
Antidepressants	Yes – 41, No - 59

**Table 4:**
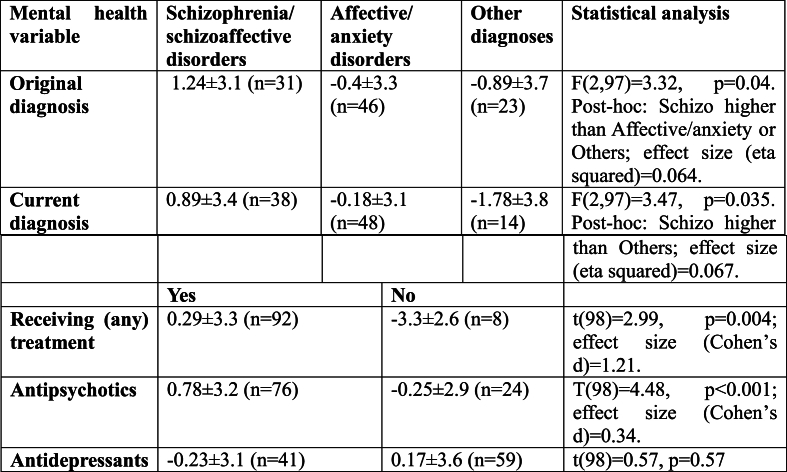
Links between mental health variables and well-being.

### 3.3 Morningness/eveningness preference

**Fig. 1:**
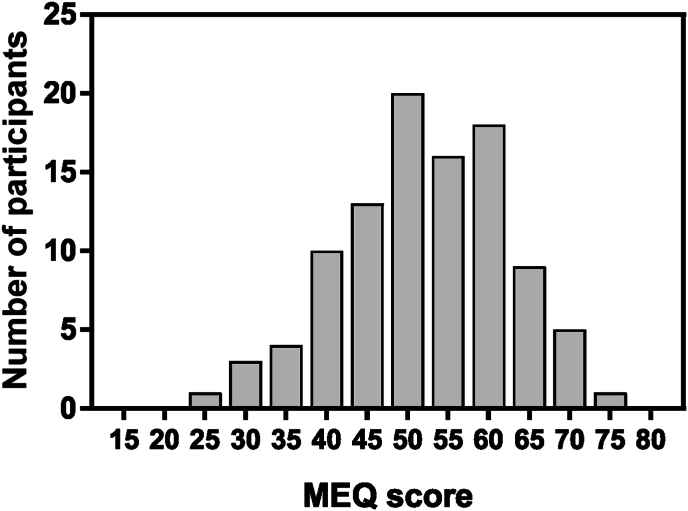
Distribution of morningness/eveningness preference (MEQ questionnaire) in the study population. Statistical analysis indicates a normal distribution [Shapiro-Wilk test: W = 0.99, p = 0.68].

MEQ shows normal distribution in the study population (Fig. 1) with 21 morning types, 58 intermediate type and 21 evening type.

**Fig. 2:**
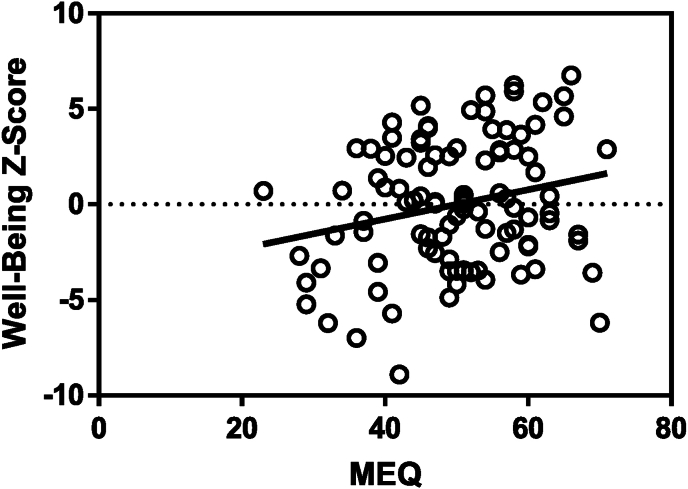
Morningness/eveningness preference is significantly correlated with combined well-being measure [Pearson's correlation, r = 0.23, p = 0.02].

Data show a significant correlation between Morningness/Eveningness preference and the combined emotional well-being variable [Fig. 2, r = 0.23, p = 0.02]. Moreover, data demonstrate a significant correlation between MEQ and 3 of the 4 measures that compound the emotional well-being construct: QoL (r = 0.24, p = 0.018), STAY (r = −0.23, p = 0.02, and CES-D (r = −0.25, p = 0.01). No correlation between MEQ and the 4th component, RASS, is demonstrated (r = 0.06, p = 0.52).

The initial analysis presented above suggests that four different variables might have a significant contribution to the combined emotional well-being outcome: (1) general health in the last month, (2) psychiatric diagnosis, (3) receiving any treatment, and (4) morningness/eveningness preference (MEQ). Original diagnosis and antipsychotics treatment are also indicated as possible contributors to emotional well-being but because both are strongly associated with the current diagnosis variable, these measures cannot be defined as independent variables.

To explore the relative contribution of these 4 factors to emotional well-being we conducted a stepwise regression analysis with the combined emotional well-being Z-score as a dependent variable and ‘general health last month”, “psychiatric diagnosis”, “receiving any treatment” and “morningness/eveningness preference” as independent factors. The regression model was significant [r = 0.45, F (4,95) = 6.05, p < 0.001] and indicated a significant contribution to emotional well-being by “psychiatric diagnosis”, “receiving any treatment”, and “morningness/eveningness preference”. The additional measure, “general health in the last month” was shown to have a near significant contribution (Table 5).

**Table 5:** **-** Regression analysis with combined emotional well-being as dependent factor.

Independent variable	Regression results
General health in the last month	r = 0.17, p = 0.078
Psychiatric diagnosis	r = −0.19, p = 0.047
Receiving any treatment	r = −0.22, p = 0.02
Morningness/eveningness preference (MEQ)	r = 0.19, p = 0.041

Altogether the results clearly demonstrate that in a cohort of psychiatric outpatients, morningness/eveningness preference is a significant contributor to a combined measure representing several domains of emotional well-being.

## 4 Discussion

The well-being and quality of life of individuals afflicted with psychiatric disorders is affected by many factors, both subjective and objective (Lehman, 1983; Levitt et al., 1990). Among these factors, many studies examined relationship between sleep parameters and well-being in general and in psychiatric patients (Jermann et al., 2022; Ritsner et al., 2004; Khurshid, 2018). Chronotypes are known to be related to duration and quality of sleep (Taillard et al., 1999) but less information is available on direct relationship between chronotypes and well-being in psychiatric patients. This is despite the data showing the effects of chronotypes on well-being in healthy individuals (Bullock, 2019; Einat et al., 2023). To this end, the current study examined the specific contribution of morningness/eveningness preference to emotional well-being in psychiatric outpatients. The results demonstrate that chronotype is a significant contributor to emotional well-being. The data suggest that chronotype independently explains approximately 4% of the variance in the emotional well-being parameter. This contribution is at the same range as factors such as the specific psychiatric diagnosis (4%) or specific treatments (5%). General health in the last month was not a statistically significant contributor in the regression analysis but showed close to significant effect (p = 0.078) with a possible partial contribution of 3%. Clearly, additional factors that were not evaluated in this study also contribute to the emotional well-being of patients.

Significant work shows the effects of chronotype on well-being in the general population (Roenneberg et al., 2003) and in specific populations such as students (Putilov et al., 2023) or shift workers (Li, 2023). Moreover, the effects of chronotype on the probability to develop psychiatric disorders or symptoms are also known (Zou et al., 2022; Kivelä et al., 2018). Here we add to that knowledge by showing the contribution of chronotypes to the well-being of psychiatric outpatients living in the community.

Unrelated to chronotypes, the well-being of psychiatric patients is known to be lower compared with healthy population (Lehman et al., 1982; Arnold et al., 2000). This difference is also demonstrated in the current study despite the design that did not include a general population sample. Comparisons between our data and general population reliable statistics in Greece show expected results for some factors related to well-being. Unemployment rate in our cohort (59/100) is significantly higher than the 12.6% unemployment rate in the general population (Bouwmans et al., 2015; O'Neill, 2023) [χ2 (1) = 23.65, p < 0.001]. Obesity is much more frequent in the study cohort (34/100) compared with the 16.7% rate (Obesity Prevalence, 2023) in the general population in Greece (Chang et al., 2021; Mazereel et al., 2020) [χ2 (1) = 4.54, p = 0.033] and the prevalence of smoking is more than double than that of the general population, 58/100 compared with 25% in the general population (Itkin et al., 2001; Kapetanstrataki et al., 2021) [χ2 (1) = 9.4, p = 0.002; ]. Interestingly, and against expectations, the rate of individuals in our sample with tertiary education (47/100) is not significantly different than the 34.6% rate in the Greek general population [χ2 (1) = 1.25, p = 0.26] (Educational attainment and labour, 2011). Also, the rate of heavy alcohol consumption in the sample is low (3/100) and corresponds with the relatively low rate (6%) in the general Greek population [χ2 (1) = 0.96, p = 0.33] (Alcohol consumption statistics, 2019). Unemployment, obesity, and smoking are factors that affect health and well-being and the current finding therefore support previous reports regarding lower well-being in psychiatric patients compared with the general population (Lehman et al., 1982; Arnold et al., 2000).

Another interesting finding, unrelated to chronotypes, is that in our cohort, individuals afflicted with schizophrenia report higher levels of subjective emotional well-being compared with the group defined with “other disorders” not schizophrenia and not affective disorders. Despite the severity of schizophrenia and its well-known effects on well-being of patients, it had been repeatedly demonstrated that appropriate treatment with antipsychotic medication is associated with higher levels of self-reported well-being (Vothknecht et al., 2011). Moreover, some of the patients categorized as “other disorders” are with a diagnosis of obsessive-compulsive disorder (OCD), and OCD patients were previously shown to have lower levels of well-being and self-reported quality of life compared with individuals afflicted with other major psychiatric disorders (Subramaniam et al., 2012).

Back to the issue of chronotypes, the emphasis in the current study is on a specific aspect of well-being, emotion. Emotional well-being is one component of well-being and is important in the context of psychiatric disorders. The demonstration here that chronotypes are significant contributor to this domain of well-being supports the general notion that circadian factors are highly involved in health and disease in general and in psychiatric disorders in particular. It had been recently proposed that circadian factors are critical in the development of the metabolic syndrome including depression (Zimmet et al., 2019). This notion is supported by demonstration that adding circadian related factors increases the possibility to predict cardiovascular diseases (Shi et al., 2021, 2022). Moreover, relatively mild circadian disturbances were demonstrated to induce depression- and anxiety-like changes in animal models (Bilu et al., 2022) and contribute to the development of depression (and possibly other psychiatric disorders) (Baron et al., 2014) as well as the response to treatment (Xavier et al., 2023; Dallaspezia et al., 2018) in humans.

Whereas the current data show direct contribution of chronotype to a composite measure of emotional well-being, no relationships were found with the specific measure for suicidality. This is in partial contrast with previous work indicating such possible relationship in the general population (Nowakowska-Domagała et al., 2023). However, the differences between general population and psychiatric patients are critical on this issue and it is highly possible that in psychiatric patients more critical factors such as diagnosis, treatment and treatment response overshadow possible contribution of chronotypes to suicidality.

In summary, the current study shows a direct contribution, small in magnitude but clearly significant, of chronotype to the emotional well-being of psychiatric outpatients. It is possible that beyond the direct contribution, chronotypes may also mediate additional factors that affect the emotional well-being of patients, but such interactions were not identified in our data. We propose that within the larger concept of the relationship between circadian rhythms and psychiatric disorders, chronotype should also be considered. We also suggest that attention to chronotypes and specific chronotherapy intervention may be of assistance to some patients.

## Funding

No specific funding was obtained for this project.

## Declaration of competing interest

The authors have no relevant financial or non-financial interests to disclose.
